# Fields or firings? Comparing the spike code and the electromagnetic field hypothesis

**DOI:** 10.3389/fpsyg.2023.1029715

**Published:** 2023-07-20

**Authors:** Tam Hunt, Mostyn Jones

**Affiliations:** ^1^Department of Psychological and Brain Sciences, University of California, Santa Barbara, CA, United States; ^2^Formerly of Washington and Jefferson College, Washington, PA, United States

**Keywords:** EM-field theories of consciousness, consciousness, spike codes, general resonance theory, ephaptic coupling, cross-frequency coupling (CFC)

## Abstract

Where is consciousness? Neurobiological theories of consciousness look primarily to synaptic firing and “spike codes” as the physical substrate of consciousness, although the specific mechanisms of consciousness remain unknown. Synaptic firing results from electrochemical processes in neuron axons and dendrites. All neurons also produce electromagnetic (EM) fields due to various mechanisms, including the electric potential created by transmembrane ion flows, known as “local field potentials,” but there are also more meso-scale and macro-scale EM fields present in the brain. The functional role of these EM fields has long been a source of debate. We suggest that these fields, in both their local and global forms, may be the primary seat of consciousness, working as a gestalt with synaptic firing and other aspects of neuroanatomy to produce the marvelous complexity of minds. We call this assertion the “electromagnetic field hypothesis.” The neuroanatomy of the brain produces the local and global EM fields but these fields are not identical with the anatomy of the brain. These fields are produced by, but not identical with, the brain, in the same manner that twigs and leaves are produced by a tree’s branches and trunk but are not the same as the branches and trunk. As such, the EM fields represent the more granular, both spatially and temporally, aspects of the brain’s structure and functioning than the neuroanatomy of the brain. The brain’s various EM fields seem to be more sensitive to small changes than the neuroanatomy of the brain. We discuss issues with the spike code approach as well as the various lines of evidence supporting our argument that the brain’s EM fields may be the primary seat of consciousness. This evidence (which occupies most of the paper) suggests that oscillating neural EM fields may make firing in neural circuits oscillate, and these oscillating circuits may help unify and guide conscious cognition.

## 1. Introduction

The conventional view in neuroscience today is that neuronal and synaptic activity are the key dynamics supporting consciousness. In other words, if we peer into the body and brain in search of the “neural correlates of consciousness” what we’ll find is that electrochemical synapse activities of various types, perhaps in particular areas of the brain, are the specific neural correlates of consciousness. These synaptic activities are, in this view, necessary and sufficient for consciousness (e.g., Crick and Koch, 1990; Li and Tsien, 2017; Humphries, 2020). We will call this “the spike code approach” or “spike code view” from now on.

But what if the spike code approach overlooks key features of the brain and consciousness? What if various spatiotemporal scales of electromagnetic (EM) fields generated by, but not identical with the anatomy of the brain, are in fact the primary seat of consciousness? In this alternative view, neurons and synaptic transmission of information are necessary for consciousness, but they are not sufficient for consciousness, at least not the complex kind that humans and other mammals (and probably other animals too) enjoy.

**Figure 1:**
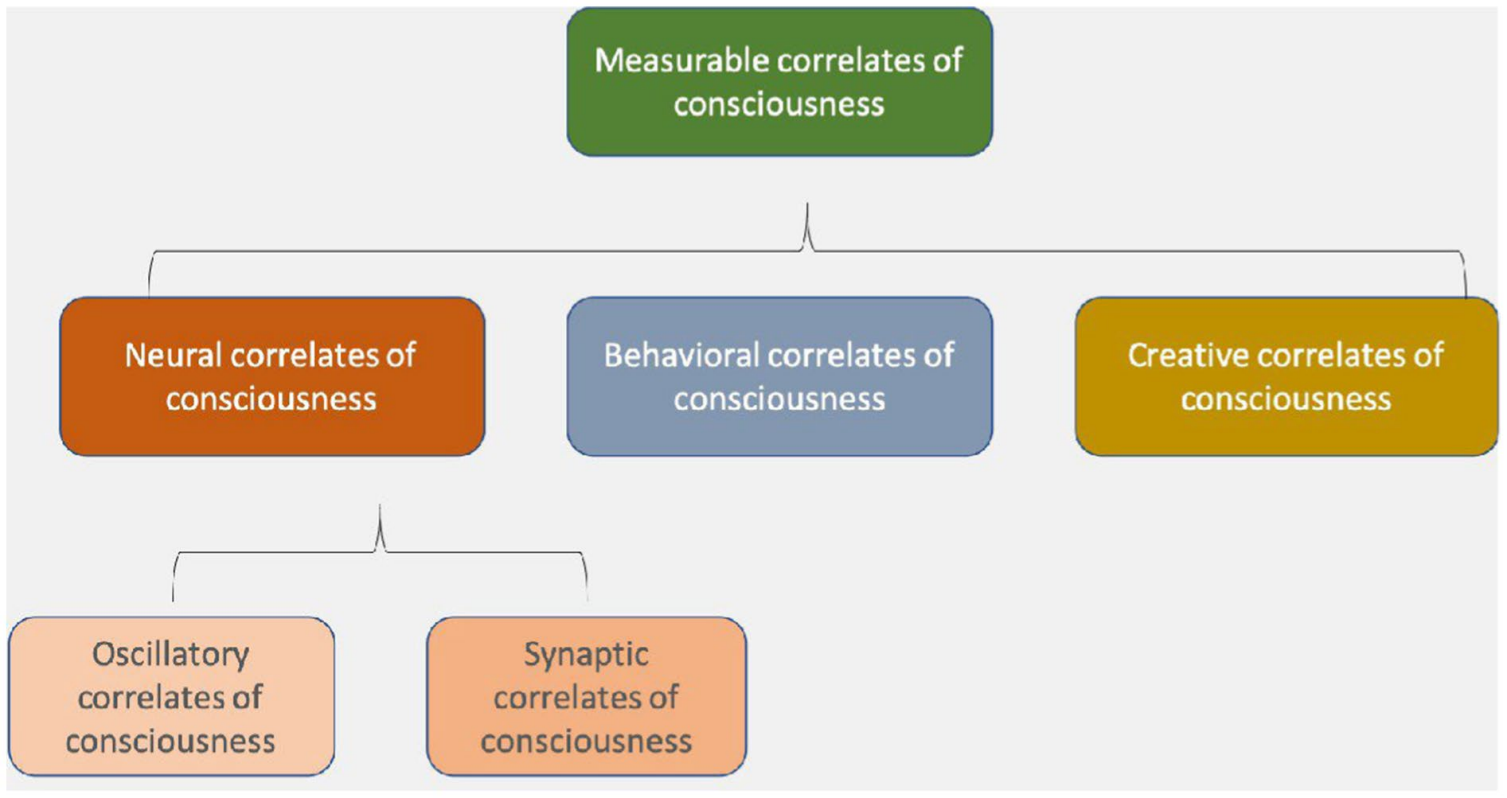
Neural correlates of consciousness include oscillatory and synaptic correlates, which are both types of electromagnetic field phenomena, at different spatial and temporal scales (Hunt et al., 2022).

Hales coined the term “electromagnetic correlates of consciousness” (EMCC) in a 2014 paper on how the brain’s endogenous (internally versus externally generated) EM fields produce consciousness (Hales, 2014). Figure 1 shows this simple taxonomy of various correlates of consciousness and suggests that the well-known “neural correlates of consciousness” should be divided into synaptic correlates and, as a new category, oscillatory or electromagnetic correlates of consciousness (Hunt et al., 2022).

If Hales and his co-thinkers like us are right, the spatially and temporally more fine-grained dynamics of local and global EM fields may be the primary seat of consciousness. Under this view, rather than looking solely for neural correlates, and their “spike codes,” we would look for specific EMCC (see Figure 1), or what we have begun calling “the resonome,” or the “oscillome,” which we define as the set of oscillating fields that create various shades of consciousness in each moment. Oscillating EM fields and synaptic dynamics, in this view, jointly comprise the neural correlates of consciousness.

This debate is highlighted in NIH researcher Douglas Fields’ 2020 book *Electric Brain* and he generally supports the view that EM fields are functionally relevant and causally potent in the brain.

We’ll examine now the various arguments in favor of each of these two approaches: (1) the spike code approach in which regional and global EM fields are largely epiphenomenal (not causally relevant to brain activity or consciousness); (2) the EM field hypothesis of consciousness, in which EM fields at all scales are not only causally relevant, but may be the primary seat of consciousness. To be clear, this EM field approach also accepts the importance of spike code dynamics in the workings of the brain and consciousness, but suggests also that there are additional EM field phenomena, working at a broader range of spatiotemporal granularity, necessary to explain the workings of consciousness.

A final prefatory note is important: the brain is *fundamentally* comprised of (almost) nothing more than EM fields (Hales, 2014; Hales and Ericson, 2022). This bears repeating: there is nothing in the brain that is not comprised fundamentally of EM fields (except, arguably, for the nucleons at the heart of each atom, comprised of strong and weak nuclear fields but which have no bearing otherwise in the physics of life).^1^ EM field dynamics simply are the physics of life and consciousness and, as such, apply to all biological structures. Accordingly, this paper is focused on the role of regional and global EM fields over and above the more localized EM fields that are, uncontroversially, the basis for neuronal and synaptic dynamics.

Our approach in this paper is to not create a new and false dichotomy between the brain, on one hand, and its EM fields on the other hand—as just explained, it’s all simply a set of nested fields. Rather, we aim to expand understanding of the EM field dynamics, which are the dynamics of both the brain and consciousness, to comprise the full range of spatiotemporal scales (local, regional and global) instead of just the highly localized dynamics of synapses.

## 2. Are the brain’s EM fields causal or epiphenomenal?

We’ll now look at representatives of these two opposing views about the role of the various EM fields in brains. We’ll also offer our own preliminary remarks about both.

Like the proverbial train whistle on a steam-powered locomotive, some scholars view the EM fields produced by the brain as perhaps only noise (epiphenomena) with no significant causal role. In this view, they do not affect the underlying function of the brain or play much role, if any, in consciousness. In response to a question about this possible causal potency of EM fields on brain functions and consciousness, a 2020 interview with Christof Koch, with one of the authors of the current paper (Hunt), is worth quoting at length because of its relevance and because Koch’s views may be seen as representative of the prevailing view among most neuroscientists.

While at this early stage of the exploration of the brain it would be foolish to categorically rule out any physical process, as an electrophysiologist I’m less enthused about ascribing specific functions to specific [EM field] frequency bands, let alone experience [Koch’s term for consciousness] for two reasons.

Firstly, by and large, the causal actors between neurons that act at the time scale relevant for consciousness (5–500 msec) are action potentials that cause, in turn, synaptic release of packets of neurotransmitters. Most neurons fire highly irregular spike trains, more compatible with a random Poisson process than with a highly synchronized, clocked process of the sort we are familiar with from electronic circuits. Yes, in a deeply asleep cortex, neuronal on–off states occur with a high degree of regularity every couple of 100 msec, leading to theta band oscillations.

**Figure 2:**
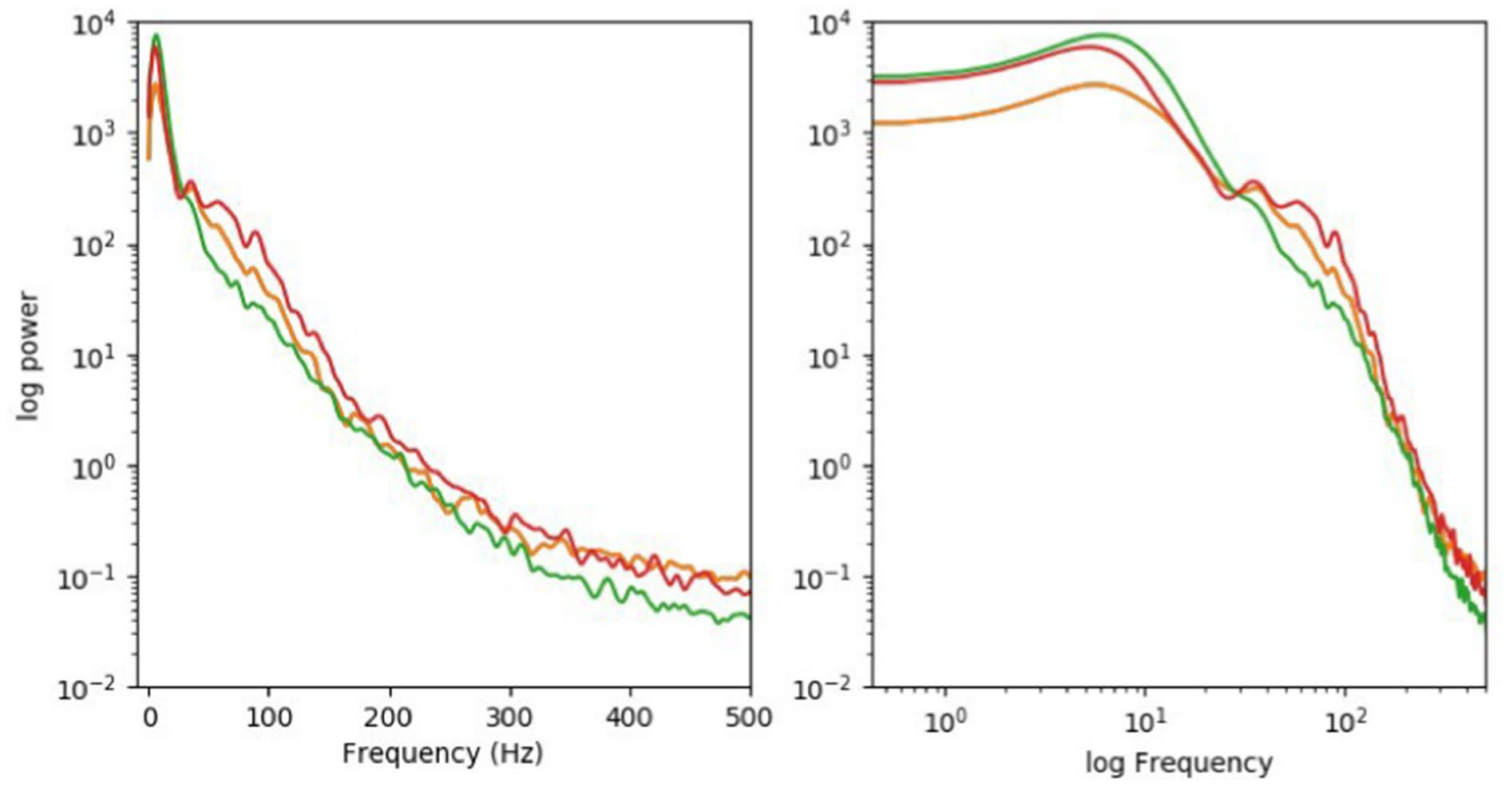
Logarithmic (right) coordinates. Typically, the EEG, picked up by large electrodes on the external scalp, will show a related spectrum. No single frequency dominates. The LFP in a human brain looks very similar.

Furthermore, given the widespread feedback nature of excitatory pyramid cells and inhibitory interneurons, certain frequencies—such as in a broadly defined gamma band extending from 30 to perhaps 100 or more Hz—can be commonly found in the awake cortex. So, yes, the EEG that is recorded from the scalp outside the skull and its sibling, the local field potential (LFP) that is recorded with thin electrodes inserted into cortex proper (through the skull), all show peaks at particular frequencies. Yet these are broad and are superimposed onto a 1/f^n type of power-law decay characteristic of many natural systems (see Figure 2 as provided by Koch).

Secondly, the extent to which oscillations in the LFP or the EEG have causal influence on firing pattern of neurons remains an open question. Consider the sounds the beating heart makes. These can be picked up by a stethoscope and can be used to diagnose cardiac conditions.

However, there is no evidence that the body exploits these sounds for any function.

My own group has provided some electrophysiological in vitro evidence that oscillations in the extracellular field at particular frequencies may be able to entrain spikes in a cell-type dependent manner (Anastassiou et al., 2011). At this point, we do not know what role such so-called ephaptic coupling (to distinguish them from the more conventional synaptic coupling) play in the human brain.

To summarize, Koch states that he is open to new evidence but he also makes it clear that he does not construe the evidence as supportive of the notion that EM fields (measured, for example, as LFPs or EEG readings) affect neuronal operations in ways sufficient to be important for consciousness, let alone being the primary seat of consciousness. Rather, Koch supports the spike code approach.

Further support for the spike code view comes from Humphries (2020). He provides a book-length overview of the science of “the spike code.” Humphries acknowledges, however, that spike activity and its relationship to consciousness remains largely unknown: “what we can predict are the new directions we want to explore. And what we want to explore is everything that is missing entirely from this book because we know nothing about them: spikes that underlie disorders of the brain, and spikes that underlie human thought processes.” He adds: “The most obvious chasm in our understanding is in all the things we did not meet on our journey from your eye to your hand. All the things of the mind I’ve not been able to tell you about, because we know so little of what spikes do to make them.”

Humphries is refreshingly humble about how little light the spike code approach currently sheds on human consciousness or consciousness more generally. It is our view that the common assumption that spike codes can explain consciousness is largely not based on data at this time. It is more of a promissory note, based on the previous success of explaining certain motor and other brain functions through the spike code.

In sketching an alternative view, that EM fields at various scales are functionally relevant and may be the primary seat of consciousness, we offer first two preliminary arguments for considering EM fields to be causally potent and possibly even the primary seat of consciousness:

1. If the brain’s endogenous EM fields were only epiphenomenal, manipulating endogenous EM fields with exogenous EM fields (TMS, tACS, TDCS, etc.) would probably not lead to changes in consciousness. The epiphenomenal view of endogenous EM fields allows only a one-way causal path: neuroanatomy producing LFPs (ECoG) and global EM fields (EEG/MEG) and no causal impact resulting from these fields back on the neuroanatomy that produced them. Just as manipulating the sound of a train’s whistle by changing its flowing steam dynamics would have no impact on the function of the locomotive that produces the steam that blows the whistle, so manipulating the brain’s EM fields with exogenous EM fields would, in this view, have no impact on consciousness. Yet we know from abundant data that there is a direct impact of various transcranial brain stimulation (TBS) techniques, as well as Deep Brain Stimulation techniques that operate from devices embedded in the brain (DBS), on consciousness. Since these tools, which include TMS, tACS, tDCS, and others, use exogenous EM fields of distinct types to achieve their effects, it would not be possible to have an impact on consciousness without the brain’s endogenous EM fields being causally potent in some manner.
2. Similarly, in physics there is a strong presumption of two-way causality. For example, in discussions about the existence and nature of the ether, in the latter part of the 19th century and early 20th century, some versions of the ether were proposed that were not causally impacted by ponderous matter, but the ether itself did exert influence on ponderous matter. Einstein, among others, critiqued this notion of the ether as “unnatural” because all other things in nature seemed to display a two-way causality (see Kostro, 2000). Einstein’s space–time, which has replaced notions of a physical ether, exhibits two-way causality. The notion of EM fields as epiphenomenal is a similarly “unnatural” view of the physics of the brain and consciousness.

These considerations are by no means dispositive of the question at issue: what role do EM fields play in consciousness? They are offered, rather as broad considerations in helping explain consciousness.

Another consideration which suggests that EM fields are conscious is the failure of purely neuronal accounts of standard neuroscience to explain how separate processing circuits bind to form our unified experience such as the unified sensory and emotional experience of seeing an old friend.

For example, some ventral-cortical detectors integrate many lower detectors to recognize particular objects, such as faces, as unified wholes. Yet there are no top-level detectors to recognize all possible visual scenes. Indeed, we can never have a top-level detector for each possible visual scene. So, while standard neuroscience has explained our vision with respect to some shapes and objects, it has not yet explained our perception of the overall unified shapes and layouts in visual images.

Another example of this binding problem is that visual processing uses separate, parallel circuits for color and shape, and it’s unclear how they combine to form complete images. Ascending color and shape circuits have few if any synapses for linking their neurons to create colored shapes. Nor do they converge on any central visual area (Zeki, 1993, p. 296; 2003, p. 216). Zeki may have overlooked feedbacks from higher cortex into lower level maps (e.g., see Kawato, 1997; Lamme, 2004; Larkum, 2013). Arguably, these feedbacks might indirectly bind color and shape. But to encode detailed images, feedbacks would have to systematically connect shape and color elements point by point all across neural maps, which even the most detailed maps fail to do (Nor is there any evidence of a central cortical area which higher cognitive functions connect into so as to account for the mind’s overall unity (*ibid.*).).

Nor does the firing of color and shape circuits in synchronized lockstep wholly encode their binding (Jones, 2017). For example, Thiele and Stoner (2003), Dong et al. (2008), and others found that neural firing synchrony does not always correlate with color and shape binding. Also, Koch et al. (2016) point out that neural firing synchrony occurs without consciousness during anesthesia and seizures. Here hypersynchrony seems to disintegrate binding. So, there is little support for binding by neural-firing synchrony. This is a different kind of synchrony than the binding by EM-field synchrony which this paper addresses, and our tentative view put forth in this paper is that it is various scales of EM field synchrony that is responsible for binding.

EM field theories of consciousness may explain binding in terms of EM fields, rather than neural-firing synchrony, without the problems above (Jones, 2017; Jones and Hunt, 2023, in progress). For example, while the brain lacks a single, central circuitry to bind colors and shapes together, its separate circuits still generate a single, continuous, unified EM field that can reach across neurons as a continuous wave, thus pooling consciousness in myriad neurons and circuits into a single, unified consciousness (This unified experience dissolves back into isolated, subliminal levels as the EM field steadily weakens). Even where circuits do not connect synaptically, they can still unite if the localized EM fields associated with their diffuse ion currents make contact, as color and shape circuits do in cortical maps.

EM fields are strongest—and most capable of unifying experiences—where they are synchronized (i.e., where their peaks and troughs reinforce rather than cancel each other out). Again, this field synchrony differs from neural-firing synchrony, although they are of course related phenomena and can affect each other. The former has the virtue of being more flexible than the latter. For this field synchrony allows different frequencies (gamma, theta, etc.) to align in phase by nesting within each other (see Section 5.1 below). This flexibility makes field synchrony more capable of explaining the binding which underlies unified, conscious cognition.

Evidence that unified cognition comes from these EM fields takes four forms. First, as already argued, neuronal connections and neural-firing synchrony seem to fail to explain the mind’s unity. Second, Koch et al. (2016) argue that locally activated EEGs actually track conscious perceptions across brains better than other events, such as neural-firing synchrony or P300 events. This EEG evidence correlates unified perceptions with EM fields in sensory areas. Third, EM fields alone—in the absence of particles or synapses—evidently propagate signals across slices in hippocampal tissue (Chiang et al., 2019). This indicates that it very likely the continuous fields that unify this activity. Fourth, as noted below, there’s growing evidence that oscillating fields help our conscious attention to control aspects of cognition. This indicates that subjects may exert forces in the form of EM fields. This is arguably a crucial facet in the unifying of conscious cognition.

Again, fields theories do not deny the crucial role of neuronal networks in contributing to consciousness. For example, the processing of binocular rivalry, color constancy, and object recognition, etc. are all vital to producing visual images. Field theories only argue that this neuronalprocessing operates behind the scenes. What is conscious in visual networks is just the EM field they generate, according to field theories.

To summarize, we have considered some or the arguments against EM fields being causally potent and we have found that these arguments do not rule out the EM field hypothesis.

## 3. EM field theories of consciousness

Of the dozens of EM-field theories of consciousness, the one that is most relevant to our account of oscillating fields in conscious cognition is the General Resonance Theory (GRT) of consciousness (Hunt, 2011, 2014, 2020; Schooler et al., 2011; Hunt and Schooler, 2019; Young et al., 2022; Hunt et al., 2022). GRT attempts to characterize the nature of consciousness and offers a quantitative framework for measuring the capacity for consciousness in any given organism or physical system.

GRT assumes that all matter is associated with at least some capacity for phenomenal consciousness, but that consciousness is extremely rudimentary in the vast majority of cases, due to a lack of physical complexity that is mirrored by a lack of mental complexity. EM fields that are associated with all baryonic matter (i.e., charged particles) are thought to be the primary seat of consciousness in GRT. The resonance (similar but not synonymous with synchronization and coherence) between various nested EM fields and the information processing afforded by EM fields are considered necessary and sufficient for consciousness. This EM field-based theory is applicable to all physical structures (of normal matter) and is not limited only to neurobiological or even biological structures (Hunt and Schooler, 2019).

Resonance is the key mechanism by which the basic constituents of consciousness combine into more complex types of consciousness. As the matter becomes more complex and integrated, the capacity for phenomenal consciousness increases. This is the case because shared resonance allows for phase transitions in the speed and bandwidth of information exchange to occur at various organizational levels, allowing previously disordered systems to self-organize and thus become coherent at multiple scales. The speed and bandwidth of information flows achieve a step change through such a phase transition, allowing for the unity of consciousness in each moment.

The spatial and temporal boundaries of any particular conscious entity are established by the slowest-frequency shared resonance within that conscious entity, for each particular information/energy pathway (Hunt, 2020; Young et al., 2022). Shared resonance and resulting resonance chains are the key mechanisms for self-organization and are constantly changing in most entities (Walleczek, 2000). Thus, the spatial and temporal boundaries of conscious entities will be constantly changing at least a little (Hunt calls this constantly changing EM field structure in human and mammalian brains “the blob” in Hunt, 2020).

Most combinations of consciousness, in which less complex entities combine into more complex entities in biological structures like mammal brains, will be comprised of a nested hierarchy of conscious entities, with one dominant conscious entity in each moment, without extinction (elimination) of the nested entities. This notion is stated well by Whitehead (1929): “The many become one and are increased by one.” This lack of extinction of subsidiary entities distinguishes GRT from IIT and other theories that assume the extinction of nested conscious entities, leaving only one macro-conscious entity left (this is a consequence of, e.g., IIT’s “exclusion principle”).

It should be noted that GRT compares in interesting ways with the Integrated World Modeling Theory (IWMT) in Safron (2020). The latter is an intriguing attempt to reconcile integrated information theory and global neuronal workspace theory within a unified systems theory. Here consciousness is “what it is like to be processes capable of generating integrated models of systems and worlds with spatial, temporal, and causal coherence” (p. 1). This involves “synchronized couplings [that] take the form of hierarchically organized modules.” These further involve “connectome harmonics” and “communication through coherence” (p. 14).

IWMT and GRT thus both seem to align at the most abstract level in that both rely on communications *via* coherent resonances between parts to produce coherent wholes. But GRT has a narrower view of which hardwares embody these mental systems. For reasons given above in Section 2 (and below at the start of Sections 4, 5), GRT attributes unified, conscious cognition to EM fields associated with neuronal circuits rather than the neuronal circuits themselves (their connections or synchronies) or to information transfers in general.

An implication of this EM approach is that it attributes minds not only to the EM fields in organic brains, but also to artificial brains that may eventually replicate these organic fields. This partly aligns GRT with Safron’s abstract account of minds based on various possible kinds of hardware.

We will focus now on the physical mechanisms by which EM fields may form the physical basis for consciousness.

## 4. Regional and global EM fields interact with neural circuits

The remainder of our paper will build on the arguments above for the EM-field hypothesis. The outline of the overall argument is as follows. (1) Conscious cognition is unified by synchronized EM fields, not only by the underlying circuits generating them (from Section 2 above). (2) These oscillating EM fields also help to make the firing of the circuits oscillate coherently (see Section 4 below). (3) Oscillating circuits guide conscious cognition (Section 5). (4) So, EM fields help guide and unify conscious cognition, which means that they aren’t epiphenomenal, and may in fact be the primary seat of consciousness (Section 6). In accordance with this outline, the present section will explain (2) above by reviewing evidence which suggests that the brain’s extra-synaptic EM fields (local, regional and global) help make neuronal circuits oscillate through increased and more synchronized synaptic spikes.

It is, again, important to note the physical fact that all parts of the brain are comprised of nothing more than EM fields, fundamentally, so what we are focused on in this section is the role of regional and global EM fields over and above the highly localized EM fields that (uncontroversially) comprise the totality of neuronal and synaptic dynamics (Hales, 2014; Hales and Ericson, 2022). These local, regional and global EM fields are produced by, but not identical with, the brain, in the same manner that twigs and leaves are produced by a tree’s branches and trunk but are not the same as the branches and trunk. As such, the EM fields represent the more granular, both spatially and temporally, aspects of the brain’s structure and functioning than the more obvious neuroanatomy of the brain. The brain’s regional and global EM fields seem to be more sensitive to small changes than the neuroanatomy of the brain, and thus may be capable of far higher rates of information processing and accompanying phenomenal consciousness. These fleeting, flexible fields may help explain how kaleidoscopic experiences emerge from relatively fixed neuronal structures much like intricate music arises from a fixed orchestra (*cf.* Fingelkurts et al., 2010). We flesh out these statements below.

### 4.1. How do neurons communicate?

Neurons and other excitable cells communicate via action potentials, i.e., rapid sequences of changes in the voltage across the cells’ membranes that propagate signals along the membranes. At least four structures or activities contribute to changes in neurons’ electrical potentials that may culminate in action potentials: (1) Synapses are specialized structures that release neurotransmitters between cells; (2) gap junctions are tiny channels that bridge adjacent cells, thus allowing charged particles (ions) to flow directly between the cells; (3) diffusion can move particles across the fluid extracellular space between cells without synapses or gap junctions; and, finally, (4) ephaptic coupling is “proposed to involve cell-to-cell transfer of electrical activation via electric fields, or ion transients” (Chiang et al., 2019; Gourdie, 2019). EEGs detect these ion currents and their fields some distance from their origins. While this involves some diffusion, the prime mover is electrical.

While communicating, neurons’ activities naturally oscillate between firing and nonfiring states, and these oscillations can sometimes align so that they fire together. This can occur due to rhythmic external stimuli such as flickering visual inputs to retinas. It can also occur due to synaptic interactions between neurons. Entrainment is this process of rhythmic stimulation (endogenous or exogenous) causing neurons to synchronize their firing.

The prevailing view has long been that EM fields are so weak in brains that they are virtually negligible in terms of any effects on brains or consciousness. But this view has changed in recent years. Looking at the evolutionary origins of EM field oscillations, Buzsaki (2004) states: “These [electromagnetic field brain] oscillations are phylogenetically preserved, suggesting that they are functionally relevant. Recent findings indicate that network oscillations bias input selection, temporally link neurons into assemblies, and facilitate synaptic plasticity, mechanisms that cooperatively support temporal representation and long-term consolidation of information.”

A series of experiments—including Frohlich and McCormick (2010), Anastassiou et al. (2011), and Anastassiou and Koch (2015)—the last was mentioned in the interview with Koch quoted above—showed that even weak exogenous (externally caused) fields can be applied to entrain spikes within slices of neural tissue. Furthermore, computer models (such as Hales, 2014) indicate that extracellular fields can synchronize network activity and alter signaling in neural networks. This growing body of data suggests that endogenous (internally caused) fields can affect rhythms in brains.

Skeptics reply that these effects of exogenous fields on neural tissue still do not show that the brain’s own endogenous fields influence neural operations. They also argue that it would be hard to show that these endogenous fields and ion currents influence neurons because this effect could be due instead to ion currents in gap junctions.

But recent experiments have countered such skepticism with evidence that ephaptic effects do occur in various tissues. This data will be detailed in the rest of this section. The studies most important to our aims are examined in some detail, while the others are only sketched.

Gourdie (2019) took aim at the long-held view that gap junctions propagate action potentials (electrical impulses) in heart cells to produce heartbeats. He contended that mounting evidence has made this unlikely. For example, genetically altered mice without gap junctions retain heart function intact. Also, bird hearts have too few gap junctions to support reliable propagations.

Gourdie drew on computational and experimental work in the last decade to argue that propagation of action potentials in the heart probably involves both gap junctions and adjacent sodium-gated channels. These channels are close enough in neighboring cells (<30 nm) to enable ephaptic (electric-field) transmission of action potentials between the cells.

Zhang et al. (2019) presented evidence of ephaptic modulation of sensory circuits at the most peripheral level. They started by noting that, in general, olfactory receptor neurons (ORNs) housed in the same sensory hair in fruit flies often inhibit each other in ways that affect perceptions and behavior. Previous studies showed that, despite the lack of direct synaptic connections, activation of one ORN suppresses the activity of its neighbor (e.g., Su et al., 2012). These inhibitions appear to help in discriminating which odors are present.

For example, in the Drosophila (fruit fly) antenna, different subtypes of olfactory receptor neurons (ORNs) reside in the same sensory hair, and these inhibit each other non-synaptically. Zhang et al.’s recordings from pairs of sensory hairs impaled by the same tungsten electrode showed that direct electrical interactions (ephaptic coupling) on their own can produce lateral inhibition between ORNs. In contrast, there were no synaptic or gap-junction connections between the receptor cells to create the inhibitions. The researchers concluded that the inhibitions are mediated ephaptically. They argued that this ephaptic activity allows more rapid peripheral processing of odor-mixtures and more elaborately patterned neural coding at higher levels.

Zhang et al. explain additionally that ephaptic interactions involve uninsulated neurons packed together. This allows their electric currents to contact each other. These types of neuronal groupings commonly occur in bundles of unmyelinated axons, such as found in mammalian retinas, olfactory nerves, and interoceptive nerves. These neuronal groupings also commonly occur in the cerebellum, which serves motor movements—thus implicating ephaptic influences in motor control. These groupings also exist in hippocampus, which serves memory consolidation (see directly below). This involves transferring fleeting short-term electrochemical memory traces into more long-term chemical storage via protein synthesis. In all these cases just listed, electrical activity alters chemical activity in neurons.

Martinez-Banaclocha (2020) went further than Zhang and her team. Citing Frohlich and McCormick (2010) and Anastassiou et al. (2011), he said that ephaptic coupling “seems higher in oriented cortical structures like neocortex and hippocampus, where pyramidal cells organize in minicolumns with well-developed layers… These particular arrangements of neurons in the cerebral cortex and hippocampus allow a parallel and radial alignment (orthogonal) of the interstitial space that has relatively low impedance to the extracellular ionic currents.”

The strongest evidence for ephaptic coupling, however, has come from Dominique Durand’s team at Case Western Reserve University. For example, Chiang et al. (2019) studied slow periodic hippocampal oscillations (<1 Hz) in mice likely related to memory consolidation during sleep. They showed that these waves synchronized the propagation in ways best explained by ephaptic coupling (They characterized this coupling as a group of neurons generating an electric field capable of activating neighboring neurons, even though such fields were thought to be too weak to do so). This surprising result was triple checked at the request of the *Journal of Physiology*.

These researchers showed that the propagation speed (0.1 m/s) across longitudinal slices of hippocampal tissue taken from mice brains was unaffected by blocking synaptic activity. To start with, they eliminated all means of transmission except ephaptic coupling by cutting entirely through a hippocampal slice, severing it into two parts. The slow wave still propagated at the same speed as in the intact hippocampus until the gap reached 400 microns. Gap junctions (which directly connect cells) and synapses cannot account for how the wave activated tissue on the far side of the cut. Transmission by ion diffusion was also precluded because it’s far too slow to account for the wave’s 0.1-m/s transmission speed. In contrast, the slow wave was blocked by an anti-electric field, thus “strongly supporting the hypothesis that these waves propagate by ephaptic coupling.”

These results indicate that the slow hippocampal waves aren’t propagated by synapses, gap junctions, or ion diffusions. Instead, the propagation is explicable ephaptically by neurons generating electric fields to activate neighboring neurons, thus generating a self-propagating wave (which even regenerates itself across gaps). The authors conclude that “a wave can propagate by endogenous electric fields, instead of synaptic transmission, by activating neighboring neurons through ephaptic coupling.”

Further experiments by Durand’s laboratory (Shivacharan et al., 2019) also studied slow periodic wave activity in longitudinal slices of rodent hippocampus. They studied epileptiform waves (induced by 4-AP, a potassium-channel blocker) quite similar to the slow waves directly above. They used similar methods and reached similar conclusions.

The authors cited various studies that show this spontaneous activity propagates at a speed (0.1 m/s) that differs from those in synaptic propagation, axonal conduction, or ion diffusion. By contrast, this speed is compatible with ephaptic coupling. So they investigated whether this slow periodic propagation could be fully accounted for in ephaptic terms.

The hippocampal slice was cut with a scalpel, and the two halves were separated to verify the cut. They found that as they increased the distance between the halves slightly, activity arriving at one side of the cut still propagated across the cut to activate neurons at the other side. This showed that “propagation goes through a cut, strongly suggesting that the mechanism of propagation involves ephaptic or electrical field coupling.”

Furthermore, consistent with purely ephaptic transmission, this propagation of the wave across the slice wasn’t precluded by pharmacological blockers, including a pharmacological blockade of electrical transmission *via* gap junctions. Importantly, applying a voltage clamp completely blocked propagation of the neuronal activity by canceling the incoming field at the cut and preventing any ephaptic effects. So the ephaptic effect was shown to be necessary for propagation across the slice.

The self-propagating nature of the wave was evident from neurons on one side of the cut recruiting neurons on the other side. Also, in a separate experiment, stimulating this hippocampus slice (with a field of the same strength as its endogenous field) produced a self-propagating wave through the intact slice.

In summary, Shivacharan et al. showed that synchronized, self-propagating slow-wave activity in rodent hippocampus tissue can jump across cuts in the tissue, strongly suggesting the propagating synchrony involves the wave’s EM field. These waves propagate at similar speeds to theta waves and may serve similar functions as a timing signal for neural plasticity without disturbing synaptic weights.

The authors warn that their findings about hippocampal slow waves do not necessarily apply to the dynamics of cortical slow waves. While their hippocampus slices had a dense laminar organization, cortical organization is more heterogeneous. Propagation in the latter is more likely synaptic, especially for long ranges to other brain regions (But compare contrary passages from Shivacharan et al., 2021 below and Zhang et al., 2019 above).

More recent experiments in Durand’s lab have shown that (a) ephaptic effects occur *in vivo* in anesthetized rats, not just in tissue taken from rat brains (Subramanian et al., 2022), and (b) ephaptic effects occur in cortical tissue, not just hippocampus tissue, suggesting that the effect is robust in brain tissue (Shivacharan et al., 2021).

Han et al. (2020) showed that ephaptic coupling occurs in the cerebellar cortex of mice, which fine tunes purposeful motor activity *via* sensory feedback. They note that climbing fibers from the inferior olive enter into this cortex, where they wrap around and synapse with the highly branching dendrites of Purkinje cells. The climbing fibers have powerful action potentials, and the Purkinje cells are tightly packed together with dendrites that are parallel to each other and very large. These two factors make this network well-suited to ephaptic coupling.

They used *in vivo* and *in vitro* (inside and outside the mice) recording techniques with sub-millisecond resolution to identify the joint activity of climbing fiber and Purkinje cells. They found that a climbing fiber’s powerful action potential generates large ion currents that spill over into intercellular space and exert a huge negative electric field strong enough to ephaptically affect the excitability of cells nearly 60 microns away. This hyperpolarized nearby Purkinje cells and reduced their firing.

A single climbing fiber could thereby ephaptically and synchronously pause the firing of over a hundred Purkinje cells for several milliseconds. This yields rapid, precisely timed spiking in downstream neurons far faster than any synaptic transmission. It enables climbing fibers to play central roles in controlling cerebellar activity and learning.

They discounted any role for gap junctions in the coordinated Purkinje cell activity, for there’s no real evidence of gap junctions in these cells, and gap-junction blockers do not affect the Purkinje-cell activity (Han et al., 2018). They also discounted disynaptic influences (where cells synapse via an intermediate cell) because they have longer time spans than the observed ones.

The authors described contrasting kinds of ephaptic coupling. One contrast concerns how close in space the ephaptic effects occur to synapses. The authors said that most ephaptic couplings described previously involve currents between neurons near where they synapse. But some ephaptic coupling also involves currents between neurons distant from their synapses (as when currents from one Purkinje cell’s sodium channels opens sodium channels in a neighboring Purkinje cell, distant from synapses—see Han et al., 2018).

Another contrast in ephaptic coupling concerns the number of cells involved. Often many neurons can ephaptically interact to correlate their firing (Anastassiou et al., 2011). At other times, voltage-gated channels in single cells can generate substantial currents that ephaptically influence the excitability of neighboring neurons (Han et al., 2018).

How does the ephaptic coupling focused on by the authors align with these contrasts? Firstly, in terms of closeness to synapses, the powerful climbing-fiber currents they studied came from ion channels near synapses. Secondly, in terms of the numbers of neurons involved, these powerful currents from single climbing fibers influenced many nearby Purkinje cells.^2^^,^^3^

In summary, a number of recent published experiments have provided increasingly strong evidence of ephaptic field effects, which by definition do not rely on synaptic connections. At the least, this evidence supports a multi-modal gestalt of information and energy flows in the brain, resulting in our conscious experience in each moment of waking consciousness.

### 4.2. Is the evidence for ephaptic coupling on firm ground?

Despite the evidence above, skepticism about ephaptic coupling persists. For example, in the same interview mentioned above (Hunt, 2020), Koch replied to claims of ephaptic field effects presented in Chiang et al. (2019) above as follows:

As an experimentalist, I am skeptical of these claims, in particular given their statistical validity and effect size. Of course, at this point, no neuronal mechanisms, can be definitely ruled out (including exotic macroscopic quantum effects), as long as they don’t violate the laws of physics.

Chiang’s results might conceivably reflect statistical flukes, as Koch suggests, but the proliferating variety and number of ephaptic-coupling studies argue against Chiang et al.’s findings being flukes. In this interview (as already noted), Koch adds to his critique above by arguing that it’s unclear what role (if any) ephaptic coupling plays in brains.

We may respond to this criticism by reiterating the recent evidence above that ephaptic effects occur in uninsulated neurons packed together with parallel alignments in sensory circuits, hippocampus, cerebellar cortex, and neocortex. These sites are involved in perception, memory, motor control, and higher cognition.

Buzsaki et al. (2013) directly addresses Koch’s concerns about the averaging effect, and a 1/f^n distribution of EEG oscillations, noting that while there is a 1/f^n distribution over long temporal scales there are marked departures from that distribution during various function-related measurements, and is worth quoting at length (emphasis added):

Integrated over a long temporal scale, the power distribution of the various frequencies has the appearance of 1/f^n “noise”, partly reflecting the fact that slow oscillations generate large, synchronous membrane-potential fluctuations in many neurons in brain-wide networks, whereas faster oscillations are associated with smaller changes in membrane potential in a limited number of cells, that are synchronized only within a restricted neural volume. **Nonetheless, when the brain engages in specific functions such as processing sensory stimuli, directing attention to particular features, orienting in space, engaging working memory, or preparing movements, the dynamics of the involved structures changes and particular oscillation frequencies become dominant**. In these cases the frequency-power relationship deviates from the 1/f statistics, and a peak (bump) appears in the respective frequency band.

Moreover, given the remarkable results found by the Chiang team, the journal (*The Journal of Physiology*) required them to replicate their results before publishing their paper, which they did. Durand, the primary investigator on the Chiang et al. paper, was as surprised as everyone else about their results, as he told a science reporter in 2020^4^: “It was a jaw-dropping moment, for us and for every scientist we told about this so far.”

## 5. Oscillating circuits help guide cognition and consciousness

This section presents further evidence supporting our thesis that EM fields may help guide cognition and consciousness.

We have reviewed evidence above that oscillating neural EM fields make neural circuits oscillate coherently. Now, in accordance with our overall outline, we will review evidence that these oscillating circuits help guide conscious cognition (again, the more important studies will be discussed in more detail). These two lines of evidence will lead ultimately to our conclusion (in Section 6) that EM fields aren’t epiphenomenal, and may in fact be the primary seat of consciousness.

But it must be acknowledged from the start that the studies below which show that oscillating circuits help guide conscious cognition do not typically claim that EM fields are involved in this conscious cognition, as our overall argument contends. Our reply is to refer back to the key evidence above that it is EM fields that help to make circuits oscillate coherently—and that it is these fields that also unify conscious cognition, while circuits alone do not create this unity (Section 2). This is why our overall argument is that it is EM fields that help unify and guide conscious cognition and that these fields may be the primary seat of consciousness itself.

We first take note that neuronal oscillations consist of rhythmic patterns in membrane potentials and action potentials that may be detectable by EEGs, and other tools; (these other tools are important due to the poor spatial resolution of EEGs, as noted below) and are created by neuronal interactions. The various frequencies of the oscillations are associated with various cognitive functions.

For example, delta waves (0.1–3 Hz) are associated with dreamless, slow-wave sleep and memory formation (Huber et al., 2004). Theta waves (4–7 Hz) associate with relaxed daydreaming and are likely involved in spatial learning and navigation (Buzsaki, 2005). These waves associate with gamma activity during memory tasks (Nyhus and Curran, 2010). Alpha waves (8–12 Hz) also associate with relaxed reflective states (Roohi-Azizi et al., 2017). They may inhibit cortical areas that aren’t in use, and they may play active roles in network coordination (Palva and Palva, 2007). They also help modulate conscious perception (Gallotto et al., 2017). They may work in a top-down fashion along with beta waves to control gamma-wave activities (Fries, 2005).

At higher frequencies and more active cognitive states, Beta waves (12–38 Hz) associate with active concentration (Baumeister et al., 2008), learned rules and abstract categorization (Wutz et al., 2018), and inhibition of unneeded cortical activity (Lundvist et al., 2016). Gamma waves (38–100 Hz) associate with bottom-up roles in memory, attention, working memory, and perceptual grouping, often along with top-down theta (Buzsaki, 2006) and alpha/beta control.

### 5.1. The role of cross-frequency coupling (CFC) in cognition and consciousness

There are three main features of any EEG sine wave signal: frequency, amplitude, and an additional phase term defining the specific phase at origin. Phase synchrony, also known as phase–phase coupling, is one type of CFC that is functionally relevant for the workings of the brain and thus for consciousness (Siebenhuhner et al., 2020: “Phase synchronization of neuronal oscillations in specific frequency bands coordinates anatomically distributed neuronal processing and communication.”) Numerous theories highlight the role of synchrony, coupling, coherence, or resonance (all similar albeit not synonymous terms), as a key mechanism for brain function and thus consciousness.

For example, Crick and Koch featured this concept in their neurobiological theory of consciousness (Crick and Koch, 1990; Koch, 2004). John (2001) makes “zero phase lag synchronization” central to his electromagnetic field theory of consciousness. Varela and colleagues suggested that the most plausible candidate for large-scale integration of consciousness is the “formation of dynamic links mediated by synchrony” (Varela et al., 2001). Fries (2005, 2015) has made the concept of “communication through coherence” (neural synchrony/resonance) even more widely known. Nunez and Srinivasan (2010) have developed a “binding by resonance” approach in various works; Dehaene, 2014 highlights the role of long-range synchrony between cortical areas as a key “signature of consciousness,” (as does Koch, 2004). Hahn et al. (2014) have developed a “communication through resonance” theory of neuronal network dynamics. Grossberg (2017) has developed an Adaptive Resonance Theory (ART) of consciousness over the last two decades and argues that “all conscious states are resonant states,” but that not all resonant states are conscious states. Bandyopadhyay (2019) has made the concept of resonance and resonance chains central to his Fractal Information Theory of consciousness.

A limitation of phase synchrony in neuronal firing is that gamma-frequency waves, for example, do not always correlate with conscious cognition, as noted in our discussion of binding above. However, phase synchrony in EM fields, more generally, tends to avoid this problem, for it’s more flexible. It allows different frequencies (gamma, theta, etc.) to fire in phase by nesting within each other. It is thus more capable of explaining the binding which underlies unified, conscious cognition, as a mechanism for selective resonance across different parts of the brain and body.

Note that while the studies below typically use neuronal synchrony to help explain conscious cognition, we nonetheless construe these studies as evidence that it is actually field synchrony, rather than only neural firing synchrony, that helps explain conscious cognition. This is plausible because neuronal synchrony helps produce more large-scale EM field synchrony. The upshot of all this is that while neuronal synchrony by itself does not seem to explain conscious cognition, it still contributes to the creation and sustaining of field synchrony, which can better explain conscious cognition as a gestalt of various spatiotemporal scales of EM fields.

We’ll now examine more specific examples of CFC in the workings of the brain and consciousness.

### 5.2. Perception and neuronal oscillations

There’s significant evidence that neuronal oscillations play roles in sensory circuits by reflecting or fostering rhythmic changes in membrane excitability that weight sensory inputs and modulate sensory detection. For example, Spaak et al. (2014) presented human subjects with rhythmic visual stimuli and found through magnetoencephalography that this entrained visual cortical activity. These alpha oscillations led to better performance in visual detection tasks, supporting claims that alpha oscillations cause temporal organization of visual perception. Similarly, Helfrich et al. (2014) found that stimulating parieto-occipital cortex with 10 Hz tACS (transcranial alternating-current stimulation) entrained activity in this cortex and modulated visual detection performance, again highlighting the role of alpha oscillation in visual perception.

Neuling et al. (2012) showed that human auditory detection thresholds depended on the phase of the brain activity (the alignment of its oscillations’ crests and troughs) that was entrained by alpha frequency transcranial direct current stimulation (tDCS). Manipulation of the brain activity’s phase led to different detection thresholds, showing that auditory perception can be modulated by oscillatory processes. Gundlach et al. (2016) found that tACS applied over human occipital areas at the alpha frequency intrinsic to that area entrained alpha oscillations and modulated perception of weak somatosensory stimuli. Samaha et al. (2020) pointed out that spontaneous neural oscillations have emerged as key predictors of variations in perceptual decisions concerning, for example, the detection and discrimination of sensory stimuli (while the fidelity of stimuli remains unchanged). They claimed in particular that the amplitude of ongoing alpha oscillations “bias sensory responses and change conscious perception.”

From studies like those above, Gallotto et al. (2017) concluded that “alpha-band (7–13 Hz) may index [indicate], or even causally support, conscious perception.” One factor that helped establish the genuinely causal over the merely indexical was the technique of showing that only rhythmic—not arhythmic—stimulation supports conscious perception (see below).

Other rhythms, in addition to alpha ones, may affect conscious perception. For example, Helfrich et al. (2017) discovered evidence that frontal top-down activity involving delta oscillations helps control posterior bottom-up alpha activity, thus selectively facilitating visual perception. Participants were asked to detect a near-threshold target after a train of stimuli was presented either at an alpha frequency or arhythmically. They found that the bottom-up alpha rhythm entrained posterior cortical activity and modulated stimuli detection. Importantly, the arhythmic activity did not do so. A top-down delta rhythm from prefrontal areas modulated this alpha activity to selectively facilitate visual perception.

Vernet et al. (2019) found evidence that beta rhythms also affect conscious perception. This is important because beta waves are tied to more active, concentrated thought than alpha waves. They noted that previous evidence from numerous sources showed that the ability to consciously acknowledge the presence of a visual target is associated with beta oscillations in cortical areas, including the frontal eye field. They investigated whether this previous evidence points to a genuine causal role in visual cognition for the beta oscillations (which are coordinated by theta oscillations linked to focal attention).

These researchers recorded EEG signals on humans performing a visual detection task (reporting whether and where a visual target appeared) while receiving transcranial magnetic stimulation (TMS) to the frontal eye field. These stimulations were either arhythmic or at rhythms natural for this cortical area. They found that the rhythmic stimulation caused frontal eye field oscillations with greater phase alignment and amplitudes than the arhythmic stimulation caused. These entrained beta oscillations correlated with increased sensory consciousness (estimated by visual detection sensitivity). This finding that the magnitude of high-beta entrainment correlates with increases in visual performance “provides evidence in favor of a causal link between high-beta oscillatory activity in the frontal eye field and visual detection.” But these results should be viewed with some caution due to the study’s heavy reliance on EEGs, which can record activity from different sources and in distorted ways.

Somer et al. (2020) showed that theta oscillations in human visual cortex can modulate visual perception. Various studies indicate that perception can be modulated by the phase of neural oscillations, especially in the theta and alpha ranges (e.g., Busch and VanRullen, 2010). This oscillatory activity can be entrained in visual cortex either by transcranial alternating current stimulation (tACS) across the visual cortex—or by periodic visual stimulation (flicker). What Somer et al. investigated was whether visual perception is modulated when this tACS and flicker are synchronized.

They found that performance on a visual matching task (where subjects picked which figures looked most similar) was significantly improved when theta frequency tACS over the visual cortex were in phase with simultaneous visual stimulus flicker, but not when the two were out of phase. So, extending previous studies on visual and auditory perception, their results support a causal role for synchronized oscillations in perception.

### 5.3. Attention and neuronal oscillations

Turning from perception to attention, there’s considerable evidence to support the argument that neuronal oscillations influence attentive processes. Fiebelkorn et al. (2018) are especially helpful here because their review paper spells out in detail how theta rhythms help organize attention processes. They draw on existing evidence that environmental sampling is a rhythmic process in which covert selective attention and overt exploratory movements are separable yet tethered to theta-band activity in the attention network (e.g., Juan et al., 2008). They point out that the fronto-parietal aspect of this network is at the nexus of sensory and motor functions. It directs these coupled processes of sensory input and exploratory movements (of eyes, whiskers, etc.).

Their review paper argues that significant evidence supports the argument that this network’s theta rhythms resolve potential sensory and motor conflicts by periodically re-weighting connections between higher brain regions and either sensory or motor regions. This rhythmic re-weighting alternately promotes either sensory sampling or shifting of exploratory movements to another location. This alternation between sampling and shifting involves theta-frequency control over, for example, enhanced sensory processes at gamma frequencies, attenuated sensory processes at alpha frequencies, and attenuated motor processes at beta frequencies.

These authors speculate that the theta alternations between sampling and shifting likely evolved because they brought flexibility to attention, allowing it to disengage and shift to new objects. This rhythmic cycling through alternative representations (rather than fully processing items simultaneously) may also be evident in, for example, working memory where multiple items are entertained together.

Bastos et al. (2018) showed how various frequencies have their own roles in attention. They recorded spiking in the frontal cortex layers of monkeys performing working memory tasks. These recordings indicated that ascending gamma-frequency oscillations are linked to sensory activity while descending beta oscillations are linked to attention’s inhibition of the gamma activity. Beta rhythms thus help sculpt the focus of attention and content of working memory which is crucial to voluntary control over behavior.

Narikiyo et al. (2020) found that the claustrum, a thin structure that connects cortical and subcortical areas, plays a role in allocating attention and synchronizing cortical activities (As already noted, this neuronal synchrony is no longer treated, by itself, as a binding mechanism for unifying experience; however, it does contribute to binding by EM fields at various spatiotemporal scales, which arguably does play such a role, as argued above). They showed how the claustrum has extensive reciprocal connections across the cortex and transmits signals not to specific areas but all across many cortical areas.

They reported that the claustrum coordinates the generation of slow waves in neocortex. They used optogenetic activation of neurons in slices of mouse claustrum to show how certain claustrum neurons silence neural activity in all layers of many cortical areas, then globally synchronize cortical activity at slow frequencies.

This shows a role of the claustrum in synchronizing inhibitory interneurons across the neocortex to coordinate brain states. It indicates that the claustrum is a major hub for synchronizing global neocortical slow-wave activity.

### 5.4. Working memory and imagination

Neural oscillations may also play a strong role in working memory. Bahramisharif et al. (2018) started with Lisman and Idiart’s (1995) proposal that working memory’s representation of multiple items (such as a phone number) uses a neural mechanism in which items are repeatedly activated in sequence by means of coupled oscillatory neural activity in theta/alpha and gamma-range.

Bahramisharif et al. offered experimental support for this proposal. They showed subjects three letters in brief sequence then asked several seconds later whether a fourth letter matched one of the three. Intracranial recordings of the subjects’ electrocorticographic activity showed that as subjects recalled the list of items, this activated theta/alpha oscillations: “Simultaneously, the brain exhibited item-specific activations of gamma activity that appeared at a theta/alpha phase corresponding to the item’s position in the sequence.” This shows how interacting cortical oscillations contribute to working memory. It’s a form of cross-frequency coupling (CFC), which is a general term for different frequencies interacting in a synchronized manner in the same brain region (Lisman and Jensen, 2013).

Kay et al. (2020) showed that theta oscillations may play a role in working memory. In this role, these oscillations are instrumental to some forms of imagination, planning, and decision making. Such activities can represent hypothetical future experiences quickly and constantly over time during escape, predation, and other demanding situations.

They used rats fitted with electrodes in their dorsal hippocampus who were seeking rewards by navigating mazes. They found that different hippocampal place cells (which are used in navigating) actually fired in constant alternation at 8 Hz as rats weighed alternative routes to take in a maze. The point is not that the rats actually thought back and forth about the alternatives many times per second, but just that the theta activity kept the images constantly alive in working memory like a tuning fork keeps sustains a pitch. This rhythmic firing of the different cells presumably enables the alternatives to remain separate in imagination while uniting them in the same decision process.

This rhythm matched the hippocampal theta frequency known to entrain hippocampal neural firing. The authors speculated that the rhythmic firing of these place cells suggests the existence of “a single common dynamical process that generates representations of hypothetical scenarios, including possible futures.” They suggest that this computational process may help shed light on the origins of imaginative activity, which is currently poorly understood.

Other studies focused on theta rhythms in the grid cells of the entorhinal cortex. These grid cells are connected to hippocampal place cells and respond to the place cells. Unlike place cells, hexagonally arranged grid cells allow navigation without landmarks (as when blindfolded) using just distance and direction. Like place cells, grid cells have roles in imagination. For grid cells represent not just spatial dimensions but also conceptual dimensions. In both cases, the grid cells produce characteristic hexagonal signals detectable by fMRIs.

For example, as subjects watched a bird silhouette with stretching and shrinking legs and neck, the hexadirectional signal appeared—as if the subjects were navigating a two-dimensional (neck and legs) bird space (Constantinescu et al., 2016). Similar, grid-like hippocampal cells seem to help us imagine social spaces with dimensions of affiliation and hierarchy (Schafer and Schiller, 2018).

The point is that grid and place cells work together and exhibit theta oscillations. Yet it’s not clear whether the theta cycle helps grid cells simultaneously imagine conceptual dimensions like they help place cells simultaneously imagine alternative routes. But it’s still possible that the theta cycle is a fundamental computational unit that the hippocampal-entorhinal system uses to imagine conceptual dimensions and alternatives. As some of these authors suggest, maps in this system may help us model relational structures ranging from the spatial to the purely conceptual, allowing our imaginations to find shortcuts and infer hidden relationships.

Riddle et al. (2020) also tried to establish a causal role for theta and alpha oscillations in working memory. Previous studies showed that working memory involves prioritizing relevant information and suppressing irrelevant information. These studies also showed that the activities are linked to theta frequency oscillations in lateral prefrontal cortex and alpha oscillations in occipito-parietal cortex, respectively (e.g., Wallis et al., 2015; de Vries et al., 2020). But many of these studies relied on EEGs whose limited spatial resolution hinders their ability to isolate causes and effects—especially compared to the precision of transcranial magnetic stimulation (TMS).

To investigate whether these links between oscillations and working memory were genuinely causal in nature, rather than only correlational, Riddle et al. set up a working-memory task that cued human subjects as to which displayed item should be attended to. The past evidence above predicted (for example) that if the task triggered the prefrontal cortical response, this response would exhibit the theta oscillations linked to this cortex.

The researchers then applied transcranial magnetic stimulation (TMS) of theta, alpha, and arhythmic frequencies to prefrontal and parietal regions (identified by functional magnetic resonance imaging, fMRI). They found that the effect of the TMS depended on whether its frequency matched the oscillations in these areas that was predicted above. For example, if the working memory task was predicted to cause theta oscillation in prefrontal cortex, and the TMS was applied there at this theta frequency, then they found that working memory performed well. But if the oscillations mismatched, then working memory did not perform well. These results (and others in their paper) provide support for causal roles for prefrontal theta oscillations and parietal alpha oscillations in the inner control of working memory.

Siebenhuhner et al. (2020) looked more broadly at this role of lower theta and alpha frequencies in coupling with higher frequencies (another example of cross-frequency coupling) to coordinate brain activities. They argued that this coupling enables various frequencies of activity in anatomically distributed areas to coordinate neuronal processing. They view the different kinds of this cross-frequency coupling as essential to large-scale coordination of activities between anterior and posterior brain areas.

Working with MEG (magnetoencephalography) and EEG (electroencephalogram) techniques, Siebenhühner et al. developed their own methods of distinguishing genuine coupling from spurious artifacts to reliably identify human brain-wide coupled networks. The strength of these large-scale networks predicted cognitive performance in a separate assessment. They drew on numerous previous studies of how theta-alpha oscillations are associated with top-down regulation of brain activities, and how beta and gamma oscillations are associated with bottom-up sensory processing, as well as how beta oscillations are associated with sensorimotor processing.

### 5.5. Long-term memory

Neuronal oscillations may also play a role in long-term memory. Lisman and Jensen (2013) examined studies of theta and gamma oscillations engaged in cross-frequency coupling in the hippocampus, which is involved in memory consolidation. They reviewed evidence from various animal species that the different spatial information in memories is represented in different gamma subcycles which are nested in the overall theta cycle. They also reviewed evidence that these frequencies and their couplings are functionally important to memory performance. They conclude that theta and gamma oscillations interact in the same brain regions (such as the hippocampus) to represent multiple items in an ordered way—and these frequencies coordinate communication between brain areas for perception and memory.

Heusser et al. (2016) started with the preexisting hypothesis (which appears at various points above) that elements in an experience are represented by neuronal assemblies firing at gamma frequencies while sequential order in the experience is represented by the specific timing of the firing with respect to theta frequency. They give evidence that, during successful episodic memory formation in humans, “items in different sequence positions exhibit relatively greater gamma power along distinct phases of a theta oscillation.” This supports claims that the memory of events relies on theta-gamma coupling.

Meyer et al. (2017) showed how retrieving certain kinds of fear memories involves modifying delta and gamma oscillations in hippocampus. Memory retrieval involves interactions of hippocampus with cortex, but it increasingly becomes more regulated by the cortex. Yet some fear memories resist this change. These memories are state-dependent, that is, they remain heavily hippocampal dependent and are best retrieved if neural states for encoding and retrieval are similar in the hippocampus. These states can be induced by activating hippocampal GABA receptors via the analgesic gaboxadol. For this activates hippocampal neurons while inhibiting cortical neurons.

These authors show that in rats conditioned by electric shocks to a fear response, gaboxadol “may cause this effect by increasing delta and reducing gamma oscillations in the hippocampus and disrupting retrieval-induced hippocampal–cortical theta coherence.” The chemical activation of GABA receptors thus alters neural oscillations which in turn affect retrieval of fear memories. In this way, fear memories “encoded in a state-dependent manner remain trapped within the region that encodes them—the hippocampus—and do not become cortically dependent with the passage of time.”

Ezzyat et al. (2018) showed that the oscillations help us perform memory tasks. They gave people lists of words to recall while electrodes monitored their lateral temporal cortex’s oscillations. A computer algorithm spotted the neural waves that appeared when the people were most likely to recall the words. When those good-performance waves were absent, the researchers filled in for them by stimulating the cortex electrically. This nudge to the waves enhanced performance. So the good-performance waves seem to be needed for recalling words.

To summarize Section 5, there is considerable evidence that neuronal oscillations help guide conscious cognition. This evidence fits alongside other evidence above that this conscious cognition is unified by EM fields, not by these neuronal circuits, and that these oscillating fields also regularly influence synaptic firing and neuronal oscillations at various scales. These lines of evidence support our overall conclusion that EM fields help guide and unify conscious cognition.

## 6. Conclusion

Most of this paper has been dedicated to reviewing evidence that oscillating EM fields help guide and unify conscious cognition. This evidence implies that EM fields aren’t epiphenomena of brain operations and are, instead, functionally relevant in various important ways. The same body of evidences, while far from conclusive at this time, suggests also that the brain’s regional and global electromagnetic fields may in fact be the primary seat of consciousness, while being produced by, but not identical with, the neuroanatomical backbone of the brain. This relationship, we suggest, is similar to a large tree with a trunk, branches, twigs and leaves. While the tree produces the twigs and leaves, the twigs and leaves have a more granular spatiotemporal structure. The brain’s various electromagnetic fields are analogous to the trees twigs and leaves and, as such, have their own causal structure over and above the neuroanatomy of the brain. While skepticism of these claims exists, evidence mounts steadily to support these claims and we look forward to further research shedding additional light.

## Data availability statement

The original contributions presented in the study are included in the article/supplementary material, further inquiries can be directed to the corresponding author.

## Author contributions

MJ wrote the first draft with support from TH and then TH and MJ iterated and revised numerous times. All authors contributed to the article and approved the submitted version.

## Conflict of interest

The authors declare that the research was conducted in the absence of any commercial or financial relationships that could be construed as a potential conflict of interest.

## Publisher’s note

All claims expressed in this article are solely those of the authors and do not necessarily represent those of their affiliated organizations, or those of the publisher, the editors and the reviewers. Any product that may be evaluated in this article, or claim that may be made by its manufacturer, is not guaranteed or endorsed by the publisher.

## Footnotes

^1^A possible exception may be found in Jones (2019), who attributes different qualia to different masses (and rest energies) of membrane proteins.

^2^Ephaptic effects may even exist in glial cells, including astroglial cells and oligodendrocytes that produce myelin sheathes around axons. Schmidt et al. (2021) calculate that in myelinated axonal fibers “only ephaptic coupling can explain the reduction of [sensory] stimulus latencies with increased stimulus intensities, as observed in many psychological experiments.” Additionally, Martinez-Banaclocha (2020) argues that the biomagnetic field effects of astroglial cells can ephaptically affect cortical communications.

^3^Ephaptic effects may also exist in muscular activity. Roth (1994) suggests that such effects may feed back onto motor neuron axons, creating reverberating loops that help in repetitive firing. Also, ephaptic coupling may play a role in neuropathic pain *via* crosstalk between damaged nerves and adjacent fibers. Cohen and Mao (2014) note here that secondary hyperalgesia “is seen in adjacent undamaged tissue owing to sensitization within the CNS… In part, this may be caused by ephaptic transmission or the expansion of receptive fields of injured nerves (or both).”

^4^Online at https://www.sciencealert.com/neuroscientists-think-they-ve-found-an-entirely-new-form-of-neural-communication.
